# Evaluation of gold mineralisation potential using AHP systems and weighted overlay analysis

**DOI:** 10.1038/s41598-024-70957-8

**Published:** 2024-09-11

**Authors:** Fahad Abubakar, Joseph Omeiza Alao, Arewa James Ogah, Rufai Ayuba, Mercy Omojo Lekdukun, Yahaya Baba, Fatihu Kabir Sadiq, Emmanuel Ejiga Samson, Abubakar Aliyu

**Affiliations:** 1 Department of Geosciences, Confluence University of Science and Technology, Osara, Kogi State Nigeria; 2 Department of Physics, Air Force Institute of Technology, Kaduna, Nigeria; 3 ZASTAL, National Space Research and Development Agency, Kano, Nigeria; 4 Department of Earth Sciences, Kogi State University, Anyigba, Nigeria; 5 Department of Soil Science, Ahmadu Bello University, Zaria, Nigeria; 6 Nigerian Geological Survey Agency, Abuja, Nigeria; 7 Department of Physics, University of Maiduguri, Maiduguri, Nigeria

**Keywords:** Airborne magnetic data, Airborne radiometric data, Gold, Mineralisation, Ilesha, Southwestern Nigeria, Geomagnetism, Geophysics

## Abstract

The demand for sustainable development goals and the absence of systematic development and organised exploration for gold has prompted this study to integrate magnetic and radiometric datasets with lithology to evaluate the gold mineralisation potential in the Ilesha schist belt. This study considers 3168.72 km^2^ of the Ilesha schist belt in southwestern Nigeria, a frontier belt for gold deposits. The high-resolution airborne magnetic and radiometric datasets were processed using enhancement techniques, including the analytical signal, lineament density, and K/Th ratio. CET grid analysis, Euler deconvolution, and analytical signal depth estimation methods were used to aid the interpretation. The spatial integration and interpolation were performed using the Analytical Hierarchy Process (AHP) and weighted overlay analytical tools within the ArcGIS environment. The dominant structural controls for potential mineralisation are ENE–WSW and ESE–WNW trends. The depth of the magnetic sources revealed by the analytical signal ranged from 63.17 to 629.47 m, while depths ranging from 47.32 to 457.22 m were obtained from Euler deconvolution. The delineated highly magnetic edge sources, dense lineaments, radiometrically highlighted alteration zones, and lithological hosts for gold mineralisation were integrated to establish the gold mineralisation potential map. The AHP deductions reveal that 10.52% of the study site is within the high mineralisation potential class, a remarkable 60.39% falls within the moderate class, a significant portion (28.86%) falls within the poor class, and 0.23% is considered unfavourable. The result was optimised by validation using known mines, with 94% (i.e., 15 out of 16 mining sites) plotting within the high mineralisation potential class. This assessment provides invaluable insight for stakeholders and policymakers to embark on gold exploration and exploitation and promote sustainable mineral development.

## Introduction

Nigeria's gold production has been largely ignored due to the country's over-reliance on crude oil, a major source of income. The country's gold production began in 1913 and reached its peak between 1933 and 1943^1^. Despite the potential, gold exploration has been hindered by a lack of funding and government reluctance^2^. The absence of systematic development and organised exploration has resulted in intense artisanal work targeting primary quartz-gold reefs and their related alluvial deposits in the Nigerian goldfields^3,4^. Recently, the global pandemic and falling oil prices have further prompted Nigeria to consider diversifying its economy into solid minerals (such as gold, iron, etc.) and reducing its reliance on oil revenue^5,6^.

Gold stands as one of the most valuable commercial mineral resources in the world^7^. In Nigeria, the schist belts are primarily linked to gold mineralisation, including the Ilesha, Egbe-Isanlu, Anka, Maru, and Zuru schist belts^8–12^. Previous studies have linked gold mineralisation to structurally controlled and hydrothermal alteration processes^9,13–15^. Since structures and hydrothermal alteration zones are of interest in gold exploration, it is necessary to delineate geologic structures that may act as a conduit for the remobilisation and reworking of mineralising fluid to concentrate the gold deposit. The geophysical method has become necessary because it measures the variations in physical properties at, beneath, or near the earth's surface^16^. The radiometric and magnetic methods use the magnetic susceptibility and radioelement variation, respectively, for detecting hydrothermal alterations and structures that favour gold mineralisation^17–23^.

Magnetic surveying is frequently used in mineral exploration to locate metalliferous deposits^24–28^ because of its ability to map hydrothermal alteration zones, shallow and deep-seated geological structures, and intrusives by measuring magnetic susceptibility's variation^29–31^. Airborne magnetic surveying helps characterise regional geology in basement terrain^25,32^. They are well-known, more rapid, less expensive, and versatile geophysical tools for mapping at depositional and regional scales^33^. Because structures, hydrothermal alterations, and porphyries are believed to be the leading global source of gold, silver, and other metalliferous deposits, high-resolution magnetic data is thought to be very suitable for mapping potential zones of gold mineralisation^34–36^.

On the contrary, the natural radioelements' surface distribution is measured using the radiometric method. The radiometric method can be used to map lithology, hydrothermal alterations, and structures that may not be revealed using other methods^20,37–41^. Furthermore, it offers a reliable and accurate way to identify the zones of hydrothermal alteration^28,42–45^. The radiometric ratio (especially K/Th) maps are the most useful markers for identifying alteration zones^46,47^. Due to the capability of the radiometric survey to delineate potential gold occurrences, several studies have been conducted, including^18,20–22,48^.

One element that is essential to efficient exploration and resource management is the spatial prediction of mineral resources. A variety of variables that influence the presence of minerals in a geologically acceptable environment can be used to make predictions^49^. As a result, estimating mineralisation potential is a spatial issue that can be addressed with several variables^50^. To focus subsequent exploration efforts on regions that show promise and where investing time and resources will yield the most outstanding results, mapping mineralisation potential is a frontier task that is frequently carried out during exploration programs^51–53^. In the Ilesha schist belt, a frontier geologic setting for gold mineralisation, critical models like the AHP in the framework of multicriteria decision analysis are suitable tools for gold mineral potential investigation^50^. The consideration of AHP in this research is to create an impartial and trustworthy mineral potential map of Nigeria's Ilesha schist belt. By comparing and assessing the importance of each exploration criterion, the study aims to reduce systemic uncertainties and better understand the subsurface geological control of gold mineralisation. It is essential to employ this approach in integrating airborne magnetic and radiometric analyses in the Ilesha schist belt, where mineralisation potential is unknown.

Several researchers have investigated the Ilesha schist belt concerning its gold mineralisation, including^54^ who used lead isotopes to study the metallogeny of the gold deposit in part of the Ilesha area. Reference^55^ assessed the geochemical features of the Osu-Amuta-Itagunmodi gold mining site within the Ilesha schist belt. Reference^50^ employed AHP to integrate geologic, aeromagnetic, and resistivity data to assess the gold mineralisation potential of a small portion of the Ilesha schist belt. Reference^56^ investigated the potential occurrence of gold mineralisation in some parts using electromagnetic data. Reference^48^ used the aerial radiometric data to map alteration zones for gold mineralisation within the Ilesha schist belt. However, the resolution of the aeromagnetic and aeroradiometric data used in these previous studies is based on the acquisition parameters adopted by the Fugro Airborne Surveys in the nationwide survey conducted between 2005 and 2010: ground terrain clearance of 80 m and a sampling interval of 500 m. Additionally, the integrated geophysical approach of gold prospecting within the Ilesha schist belt has been of small localised coverage and spatial integration of both magnetic and radiometric datasets has not been considered in this region. However, this study employs AHP systems and weighted overlay tools to integrate signally enhanced results from aerial magnetic and radiometric datasets in order to delineate the gold potential of a regional coverage of the Ilesha schist belt. This assessment utilises the high-resolution aerial magnetic and radiometric datasets by Xcalibur Airborne Geophysical acquired at 50 m terrain clearance and a 150 m sampling interval. A higher resolution like this is expected to resolve subtle changes in geology.

## Geologic settings and mineralisation

**Fig. 1:**
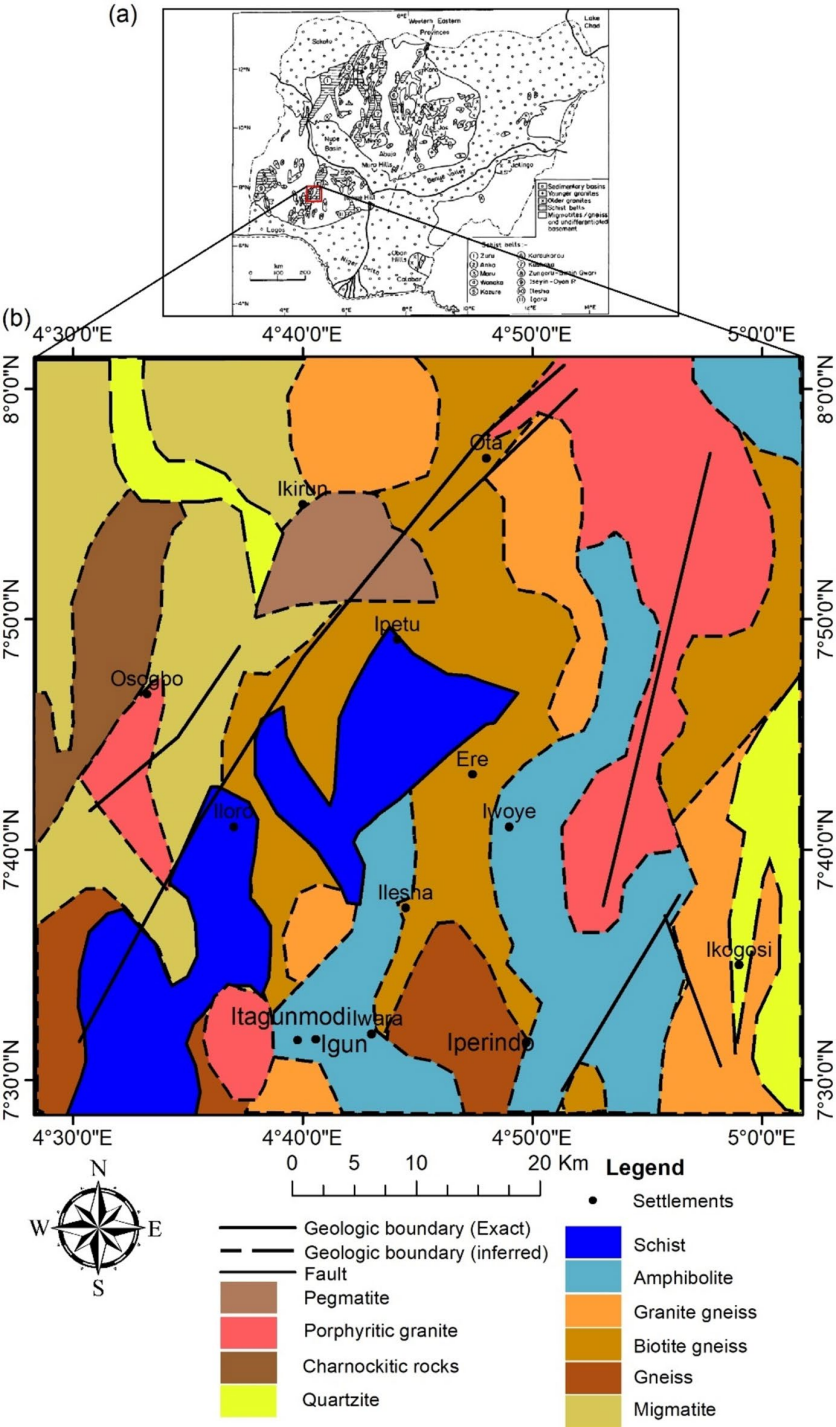
(**a**) Geology of Nigeria showing the major schist belts and the study area location (after ^10^). (**b**) Geologic map of the study site (after Ref.^57^).

**Fig. 2:**
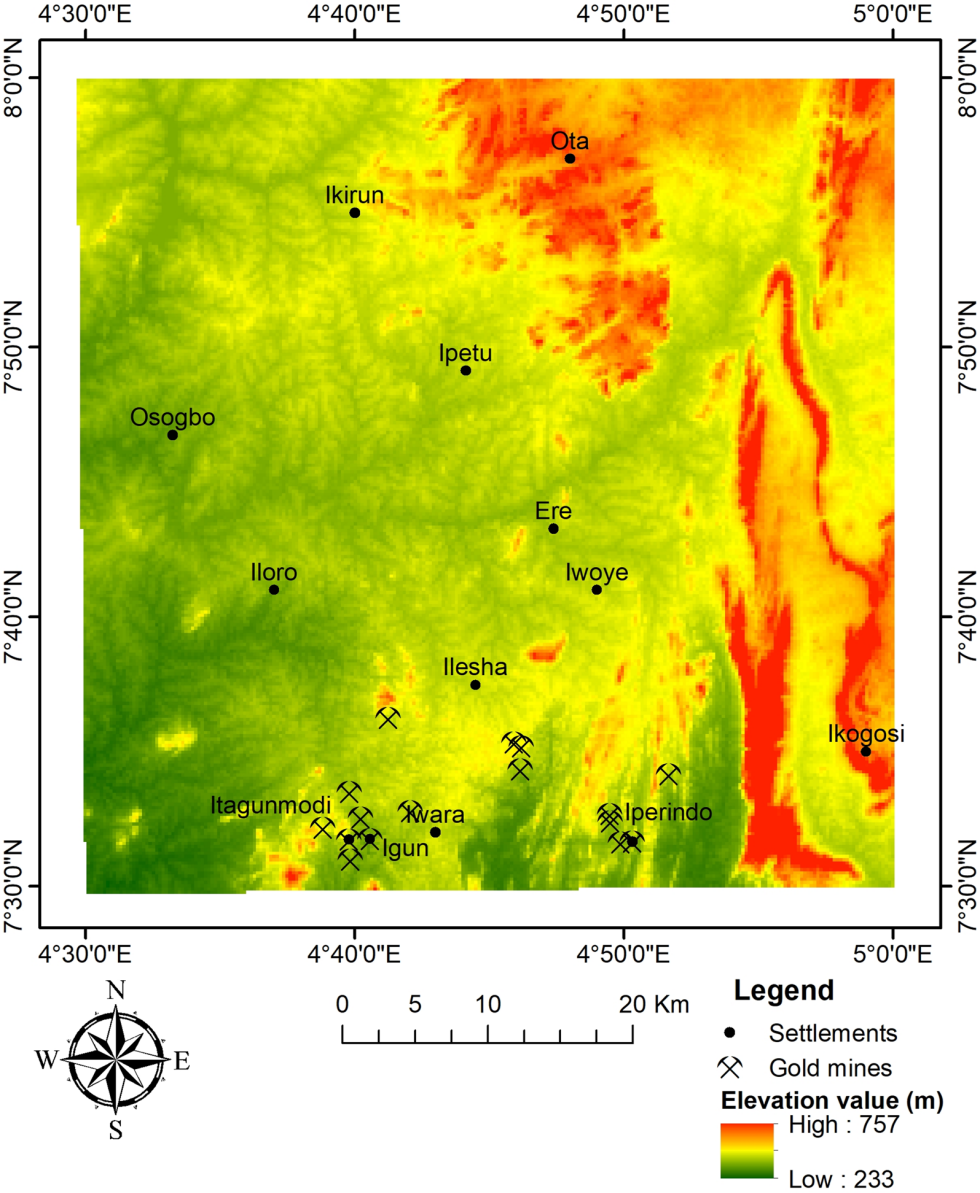
Elevation map of the study site.

The study site is situated in southwestern Nigeria, bounded by Longitude 4° 30ʹ and 5° 00ʹ E and Latitude 7° 30ʹ and 8° 00ʹ N. Its entire area is 3168.72 km^2^. It is dominated by the Ilesha schist belt (Fig. 1). The Ilesha schist belt is well known for its lode gold and alluvial mineralisation^54^. The belt stretches nearly 200 km from north to south; it widens to its most significant point in the south at 60 km. The structural unit is dominated by the NNE-SSW trending fault, which is notable for the Ife fault. The Ife fault divides the lithology into distinct units^10^. The site is dominated by amphibolite, schist, gneiss, migmatite, and granitic rocks. There are two basic gold deposits within the belt: alluvial and auriferous. Within the amphibolite, there are notable alluvial gold occurrences at Igun and Itagunmodi. The auriferous quartz veins in the granite gneiss are where the lode gold occurrences at Iperindo are located^9^. Iperindo gold is the most notable deposit within the Ilesha schist belt due to its prominence^54^. Other alluvial occurrences (artisanally mined) are distributed sporadically around these notable deposits (Fig. 2).

The gold within the belt, especially that of Iperindo, occurs with pyrite, pyrrhotite, and a lesser amount of magnetite, galena, chalcopyrite, sphalerite, and ilmenite. Its host gneiss has undergone hydrothermal alteration, and it is adjacent to the veins that contain gold^9^. Based on structural correlations, the secondary faults are primarily responsible for controlling mineralisation. The gold emplacement at Iperindo was thought to be late Pan-African^9,58,59^.

The terrain comprises lowlands and hilly areas ranging in elevation from 233 to 757 m above sea level (Fig. 2). Data from the Shuttle Radar Topography Mission (STRM) elevation, one arc-second global (30 m resolution) obtained from the USGS website (www.usgs.gov) on November 24, 2023, was used to generate the elevation map. Amphibolite and gneiss are typically found along riverbeds and exist as low-lying hills and outcrops. The gold mines are situated at shallow elevations where occurrences are found.

## Materials and methodology

**Fig. 3:**
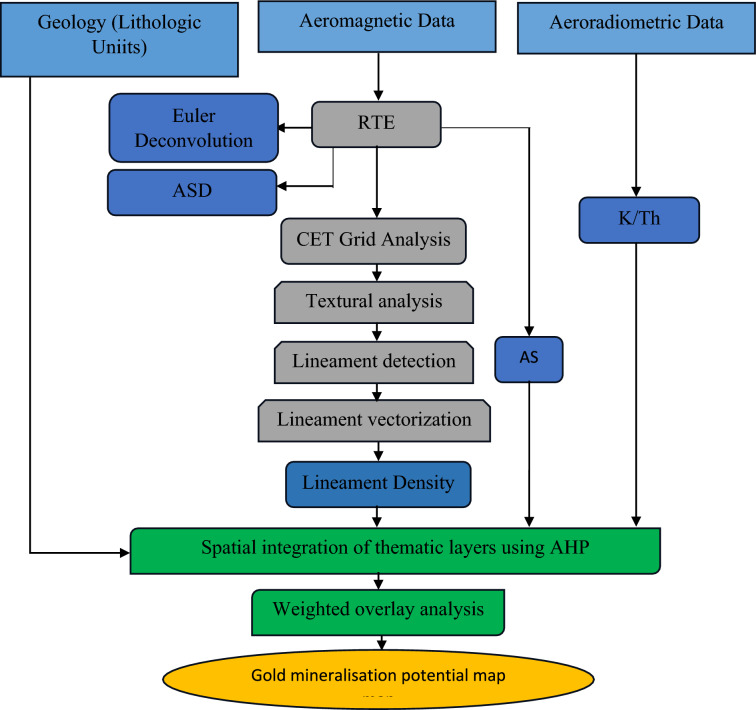
Methodological flowchart.

The airborne magnetic and radiometric datasets were obtained from the Nigerian Geological Survey Agency, Abuja, Nigeria (https://ngsa.gov.ng/). These datasets were acquired by Xcalibur Airborne Geophysical. Both the magnetic and radiometric data were collected concurrently with the following acquisition parameters: 150 m inter-profile spacing, 50 m flight height, and 1500 m tie line interval. Figure 3 shows the methodological flowchart adopted in the data analyses.

### Airborne magnetic data

**Fig. 4:**
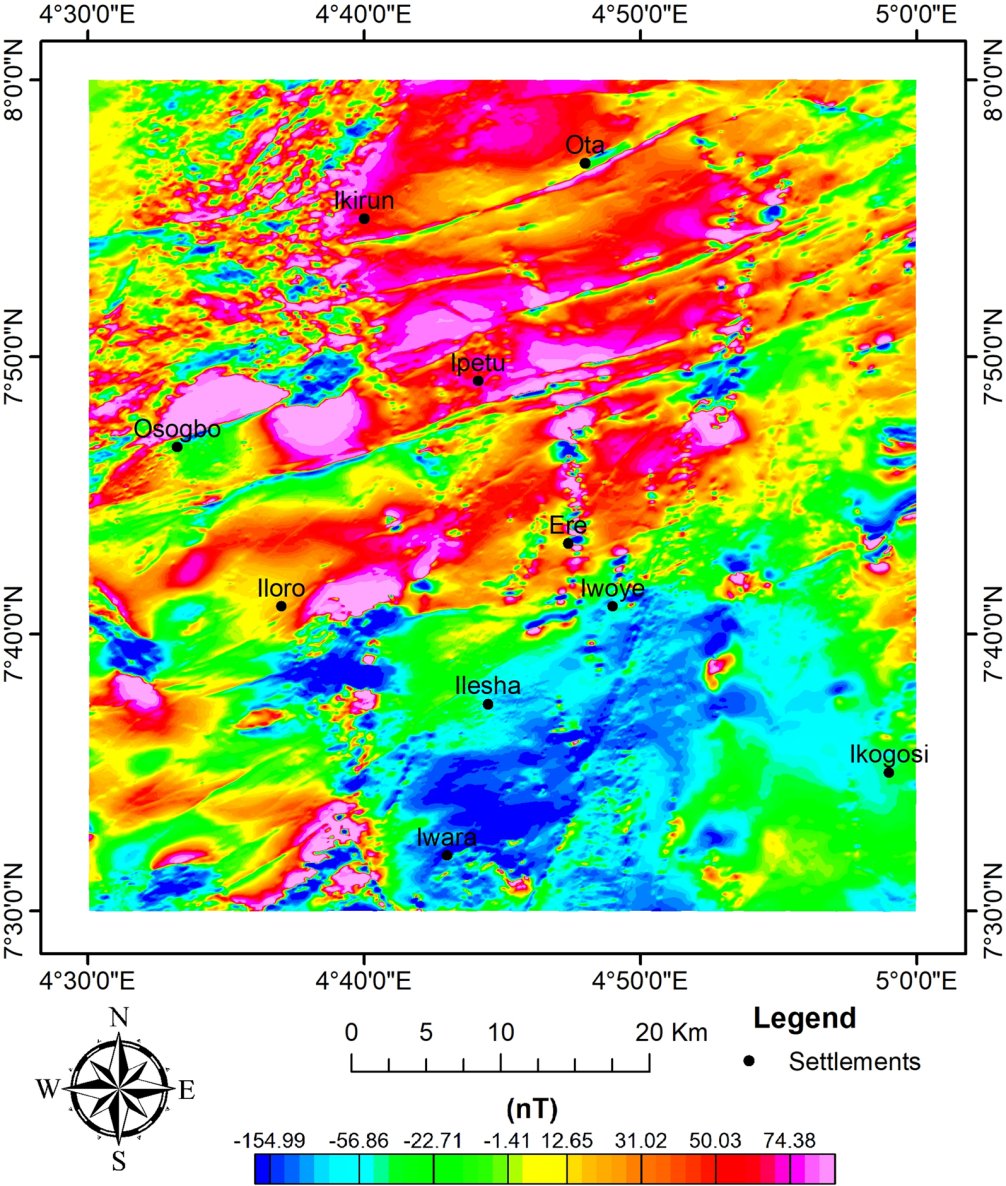
TMI map of the study site.

**Fig. 5:**
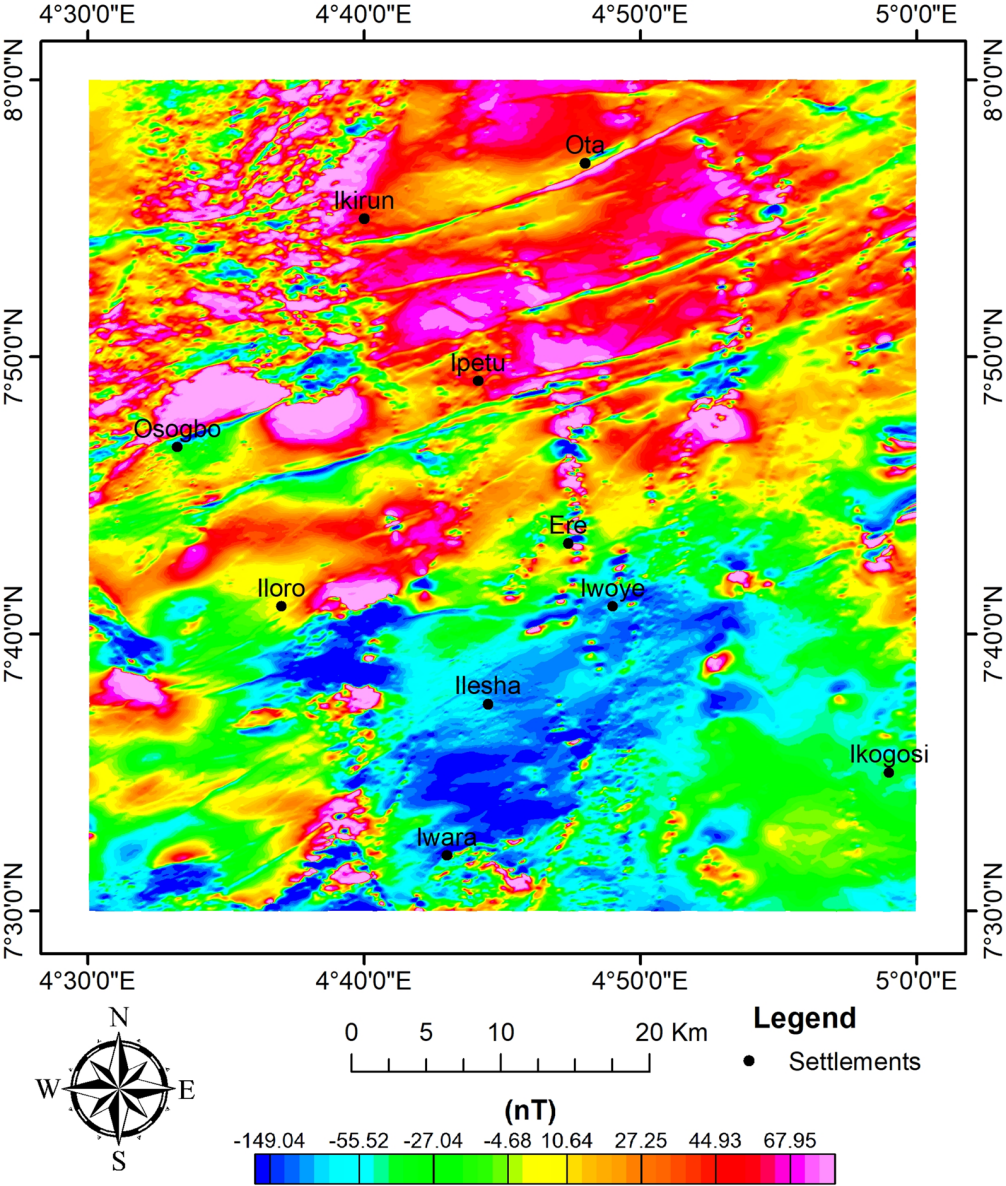
RTE map of the study site.

The airborne magnetic dataset was acquired in the form of a total magnetic intensity (TMI) grid (with the regional field of 33,000 nT removed) (Fig. 4). The geomagnetic field is expected to show variations in inclination and declination from the equator to the pole due to Nigeria's low magnetic latitude. Consequently, a reduction to the equator (RTE) modification is done to the TMI to put the maxima of magnetic strengths above their sources (Fig. 5)^60^. A magnetic inclination and declination of − 8.248 and − 0.620, respectively, were obtained from the IGRF calculator. The Geosoft program (Oasis Montaj) version 8.4 was used to process the data. Several magnetic enhancement techniques were performed to yield favourable results.

### Analytical signal and analytical signal depth analysis

The analytical signal (AS) is an enhancement technique and complex function suitable for locating subsurface magnetic structures and measuring magnetic potentials^24^. Using the evaluation of^61^, we obtain the following as the analytical signal's amplitude function:

1 $$AS\left(x,y\right)=\sqrt{{{\left(\frac{\partial MI}{\partial x}\right)}^{2}+{\left(\frac{\partial MI}{\partial y}\right)}^{2}+\left(\frac{\partial MI}{\partial z}\right)}^{2}}$$

where $MI$ is the intensity of the magnetic field. $\frac{\partial MI}{\partial x},\frac{\partial MI}{\partial y},\frac{\partial MI}{\partial z}$ is the gradient of the magnetic field intensity (in the first-order) in the *x*, *y* and *z* directions, respectively.

An analytical signal can be employed to estimate the depth to the top of magnetic causative bodies, with minimal assumptions made about the nature of the anomalies^17^. Usually, the anomalies are identified as two-dimensional magnetic source bodies^62^. The expression for the analytical signal depth (ASD) sources^63^ is given as:

2 $$ASD=\frac{AS}{{AS}_{1VG}}\times SI$$

where $AS$ is the analytical signal expressed in Eq. (1), ${AS}_{1VG}$ is the analytical signal of the first-order vertical gradient, while $SI$ is the structural index geometry of the magnetic sources, where $SI$ can be 1, 2, 3, or 4 representing contacts, dike, pipe, and sphere, respectively^64^. Other depth estimation methods, such as radially averaged power spectrum (RAPS) and Euler deconvolution, were also used.

### Euler deconvolution

The apparent depth of a magnetic source can be estimated using Euler's homogeneity equation (Euler deconvolution, Eq. (3))^65^. With the use of a structural index (SI) to represent the degree of homogeneity, the equation links the magnetic field and its derivative components to the locations of the causal sources^66^. The SI varies from 0.0 to 3.0 in general potential fields. In the magnetic field, 0, 1, 2, and 3 represent contacts, dyke or sill, pipe, and sphere, respectively. The equation below is the mathematical expression for Euler's homogeneity^66,67^.

3 $$\left(x-{x}_{0}\right)\frac{\partial MI}{\partial x}+\left(y-{y}_{0}\right)\frac{\partial MI}{\partial y}\left(z-{z}_{0}\right)\frac{\partial MI}{\partial z}=SI\left(B-MI\right)$$

where ${x}_{0}$, ${y}_{0}$, ${z}_{0}$ stands for the magnetic source's position, $SI$ is the structural index, and $B$ is the regional field of magnetism.

The minimum curvature griding algorithm was used to interpolate the depth solutions after Euler's homogeneity equation was applied to the magnetic field. This was done to produce a map of the depth source distribution. The RAPS was employed to checkmate the depth estimates from both ASD and the Euler deconvolution. The theoretical details of the RAPS model can be found in^68^.

### Centre for Exploration Target grid analysis and lineament density

The CET grid analysis (CETGA) is utilised for textural analysis and lineament delineation. It reveals all the structural controls connected to the magnetic data^69^. The details of the theoretical background of this analytical technique can be found in Refs.^69,70^. The lineament features extracted from the data were used to produce a lineament density (LD) distribution in the ArcGIS environment (ver 10.5). The LD is suitable for the exploration of minerals^39,71^. Additionally, to ascertain the dominant distribution of structural trends, the extracted lineaments were plotted on a rose diagram.

### Airborne radiometric data

The radiometric method of data acquisition was performed to quantify the naturally occurring radioactivity of gamma rays. These rays come from uranium, thorium, and potassium radioactive isotope-containing earth materials^16^. The high-resolution airborne radiometric dataset can be used to detect hydrothermal alterations, delineate radioactive geologic deposits, and map petrologic units^26,72^. Radioelemental ratios (e.g., K/Th, U/Th, U/K) are essential analytical tools suitable for identifying hydrothermal alterations^73^.

The radiometric dataset was acquired in the form of three grids: potassium (K%), uranium (eU in ppm), and thorium (eTh in ppm). This study employs K/Th to delineate the hydrothermal alteration zones.

### Gold mineralisation potential assessment

The assessment of gold mineralisation potential was carried out based on the factors that favour its delineation within the geologic settings under consideration. The gold mineralisation of the area is structurally controlled, occurs with metallic deposits, and its host is hydrothermally altered^9,54^. As a result, four parameters were adopted to determine the potential of gold mineralisation. These are lithology, analytical signal, lineament density, and K/Th ratio. The degree of limits to the specific requirements listed in Table 1 was taken into consideration when conducting the assessment of the gold mineralisation potential. By the standard deviation of the distribution of the individual parameters, the areas were categorised into highly favourable (HF), moderately favourable (MF), poorly favourable (PF), and not favourable (NF).

**Table 1:** Criteria adopted for the utilised parameters in the Ilesha schist belt.

Parameters	Highly favourable (HF)	Moderately favourable (MF)	Poorly favourable (PF)	Not favourable (NF)
Analytical signal (nT/m)	0.20–0.60	0.10—0.20	0.04–0.10	0.00–0.04
Lineament density (m/km^2^)	1.75–2.40	1.18–1.75	0.61–1.18	0.00–0.61
K/Th (%/ppm)	0.07–0.23	0.03–0.07	0.02–0.03	0.00–0.02

The raster layers of the aforementioned parameters were then transferred to the ArcGIS environment for further analysis. Units deemed to be targets for gold occurrences (i.e., using mine sites as control and expert opinions) of gold mineralisation potential were assigned to differentiate the features representing the raster data in order to perform reclassification. The standard deviation of targets was used as the basis for the classification. With the aid of the reclassification tool in ArcGIS 10.5, the features were transformed into reclassified raster layers (Fig. 13).

### AHP

The analytical hierarchy process (AHP) is a well-known and often-used method for classifying variables arranged hierarchically. It is a multicriteria analysis method that enables users to choose the parameters' weights while addressing multicriteria problems. In this study, weight values were assigned, criteria were compared using expert judgements, and the AHP model was then utilised to process the weight values^74–76^. Reference^75^ created the AHP model, which enables comparison of the relative weights of each criterion using a scale ranging from one to nine, which reports the relative ranking of each variable (Table 2). The weights of the pairwise comparison matrix priorities were then established.

**Table 2:** Fundamental scale used in AHP pairwise comparisons.

Level of significance	The rationale for the score
1	Two characteristics of equal significance
3	Low priority is given to one over the other
5	Essential or highly significant differences between two judgements
7	A very significant difference between two judgements
9	An extreme preference for one judgement over the other
2, 4, 6, 8	Values in the middle of the two adjacent judgements
½, 1/3, ¼, … 1/9	These are the corresponding reciprocals of significance levels

The consistency ratio (CR) was computed to assess the consistency of the pairwise comparisons^77^. Equation (5) was used to calculate the CR, with values ranging from 0 to 1 following^75,76^. Desirable consistency is indicated by a CR score of less than 0.1. The consistency index (CI) is essential to assess gold mineralisation potential, and it is computed using Eq. (4).

4 $$CI=\frac{{\lambda }_{max}-d}{d-1}$$

where ${\lambda }_{max}$ is the pairwise comparison matrix's largest eigenvalue, and $d$ is the dimension of the matrix. CR is given as:

5 $$CR=\frac{CI}{RI}$$

where RI denotes the mean random consistency index.

## Results and discussion

### Airborne magnetic data

#### AS and ASD

**Fig. 6:**
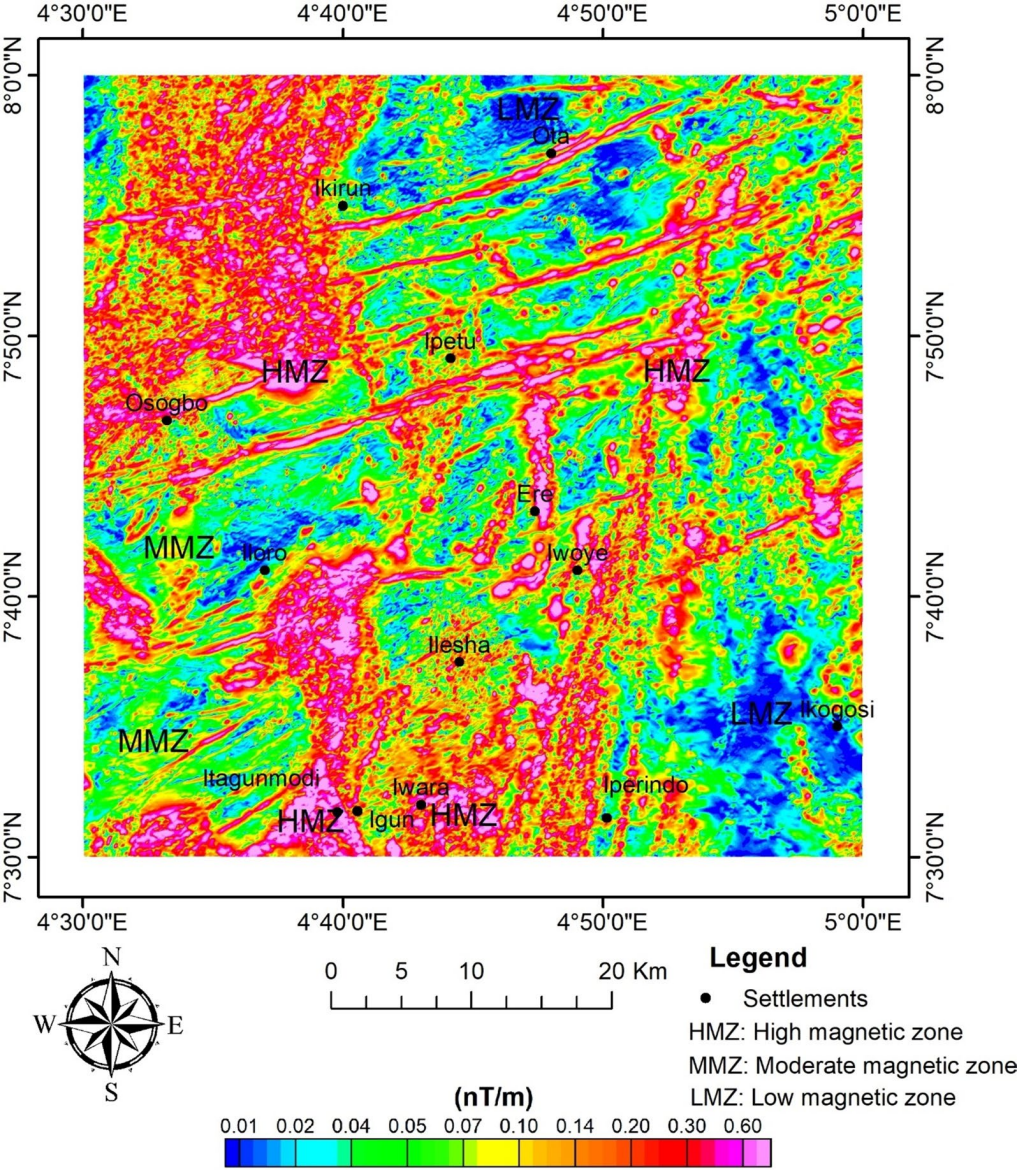
AS map of the study site.

The AS map shows the distribution of the magnetic zones; as a result, the areas were classified into high, intermediate, and low magnetic zones, with a corresponding range of 0.2–0.6 nT/m, 0.04–0.2 nT/m, and 0.01–0.04 nT/m, respectively (Fig. 6). The zones of higher maxima are mostly trending NE–SW. These zones could be representations of structures, contacts, and intrusives that could be promising targets for mineralisation^23,27^. The high and intermediate zones of magnetism can be associated with granitic rocks and very high-grade metamorphic rocks (such as migmatite, amphibolite, and granite gneiss) that contain ferromagnesian minerals^49,78^. Meanwhile, the low magnetic zones are rocks made up of more felsic minerals^16^. In addition, the structures of magnetic discontinuities that could potentially be targeted for mineral prospecting are depicted on the map.

**Fig. 7:**
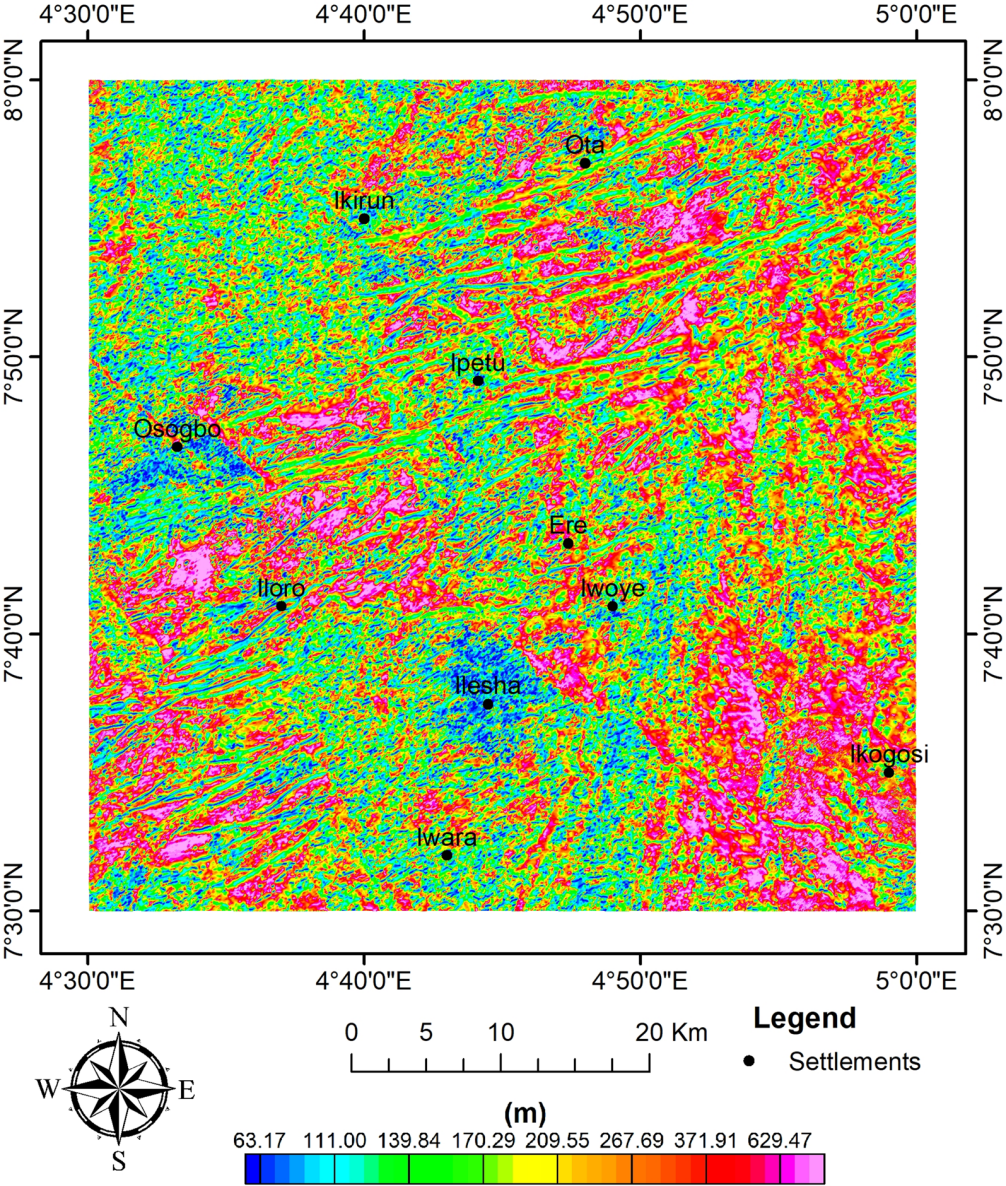
AS depth of magnetic sources.

**Fig. 8:**
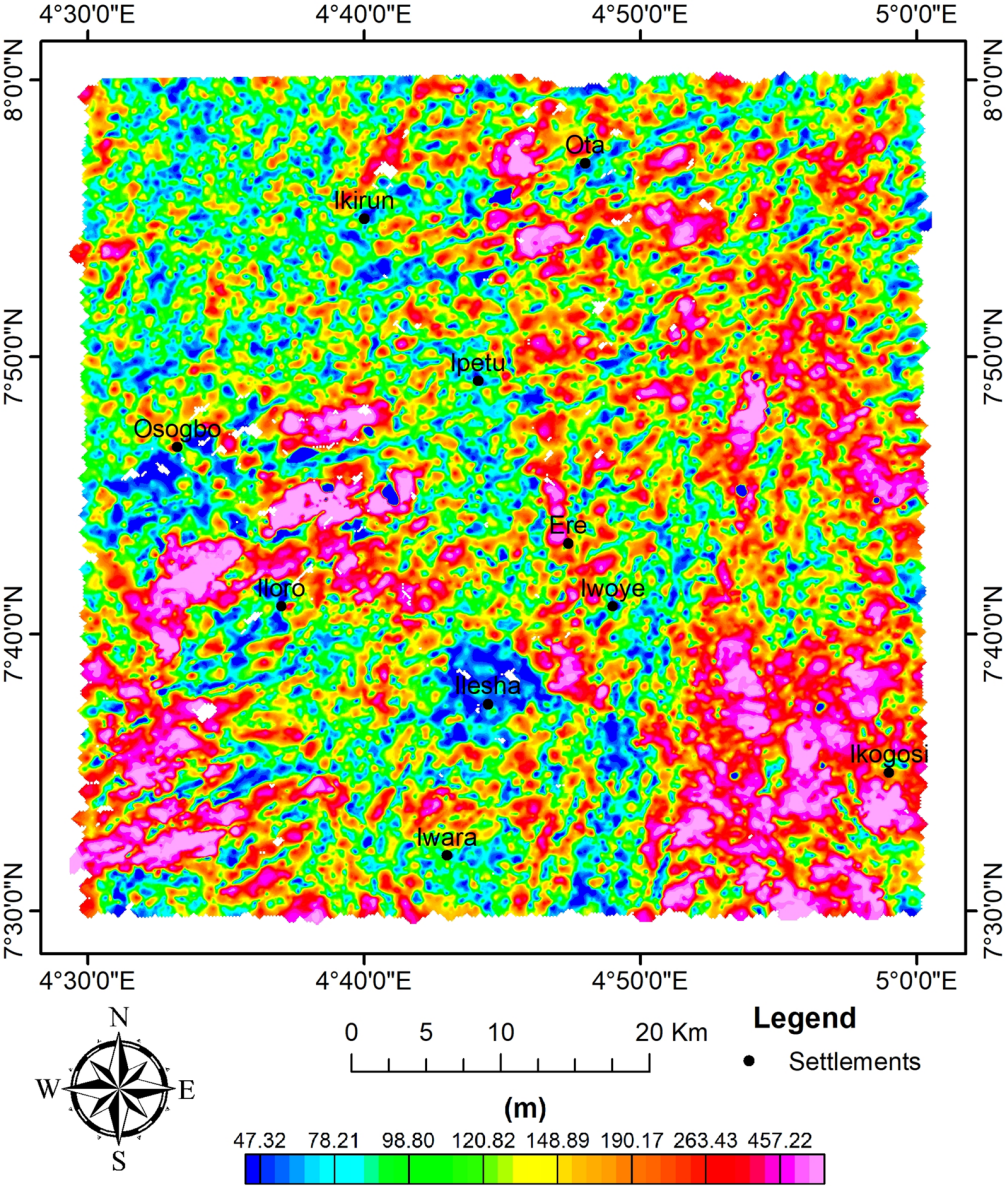
Euler depth plot of magnetic sources.

Furthermore, the method of^63,79^ was used by applying Eq. (2) to the RTE data in order to determine the depth distribution of magnetic sources (Fig. 7). As a result of the discordant magneto-structural features observed in Fig. 5, an N value of 2 for the dyke-like feature was chosen. The analytical signal's depth estimates varied from 63.17 to 629.47 m. Upon using SI of 1 (i.e., dyke-like features), Euler's deconvolution depth estimates of magnetic sources range from 47.32 to 457.22 m (Fig. 8). This shows some level of correlation with ASD.

#### CETGA and LD

**Fig. 9:**
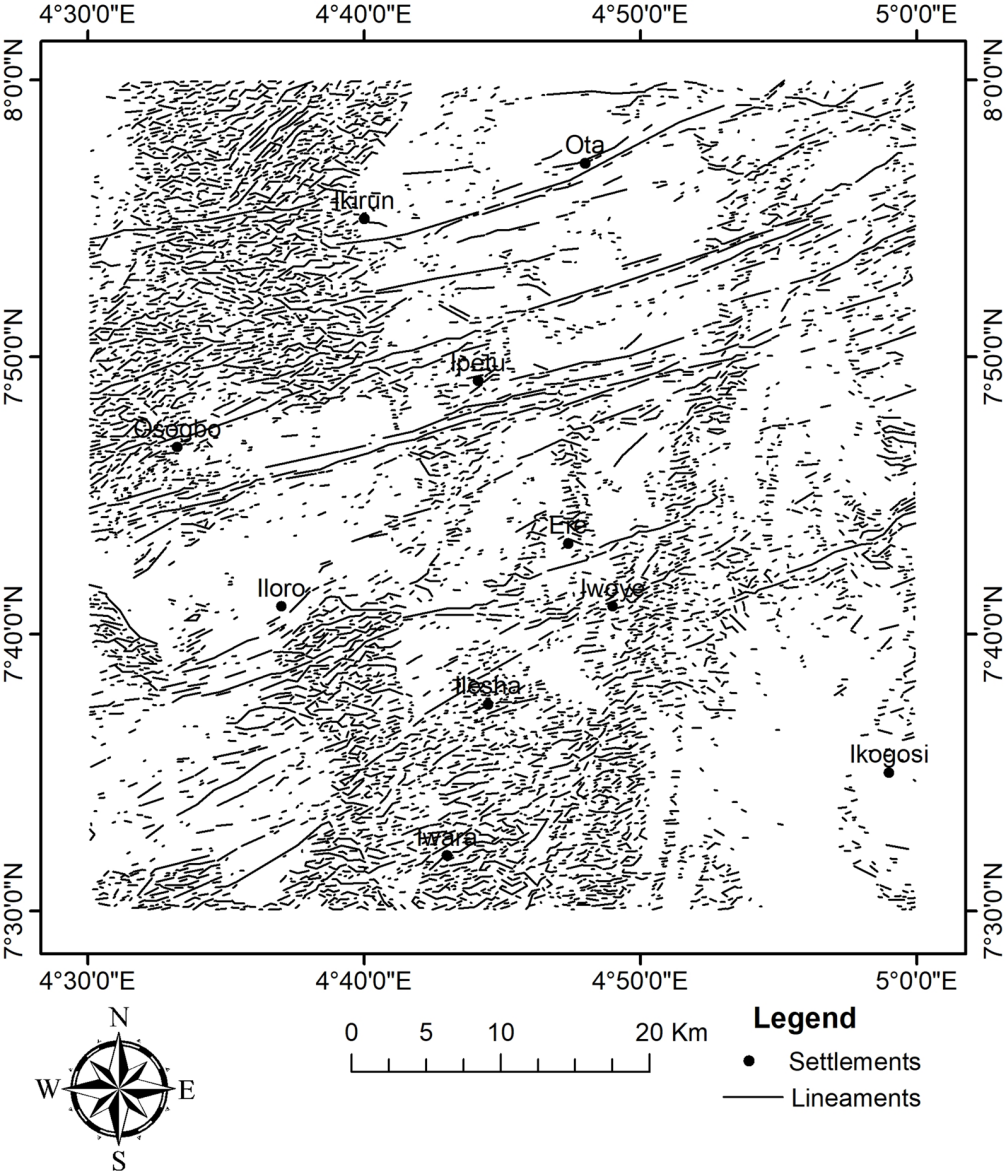
Lineament map of the study site.

**Fig. 10:**
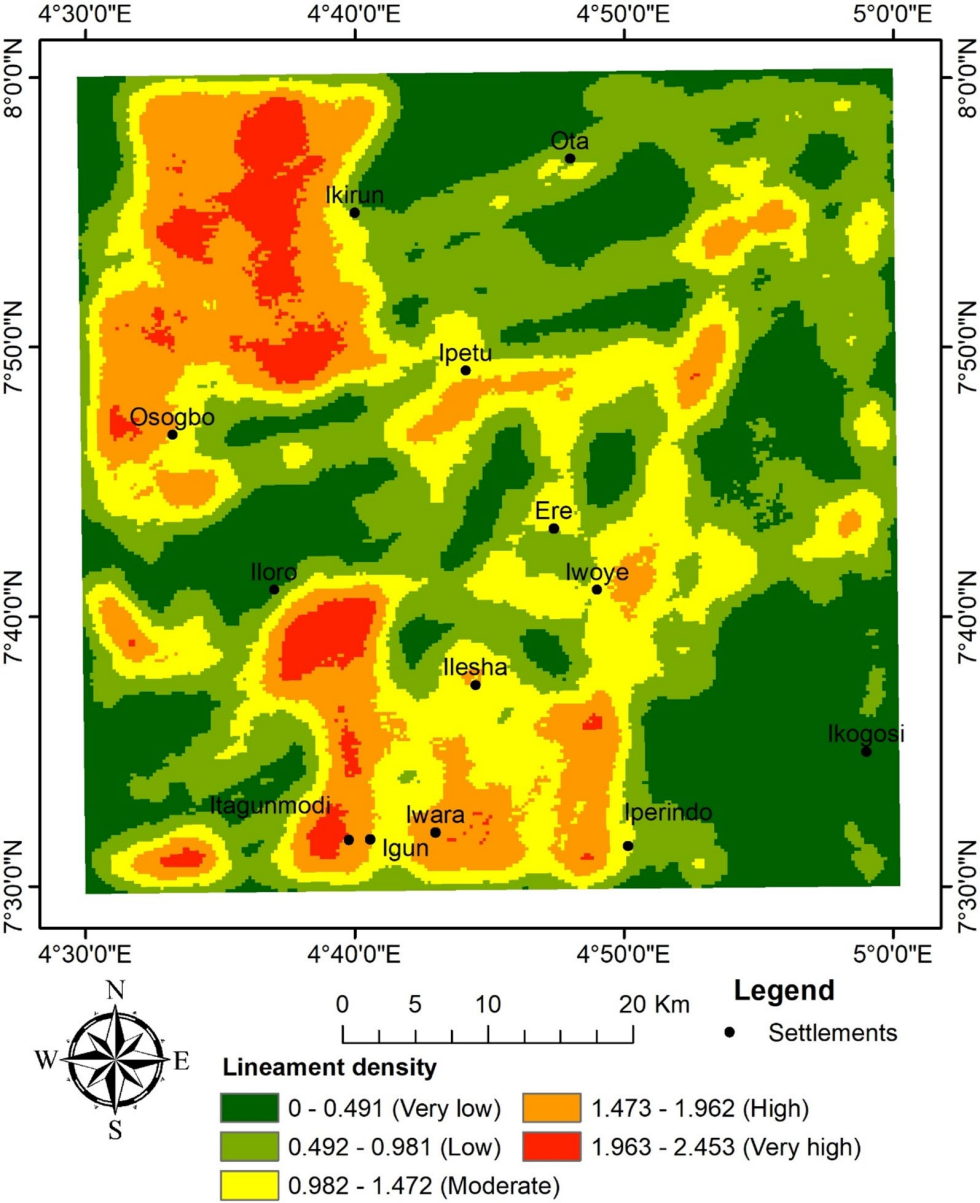
Lineament density map of the study site.

**Fig. 11:**
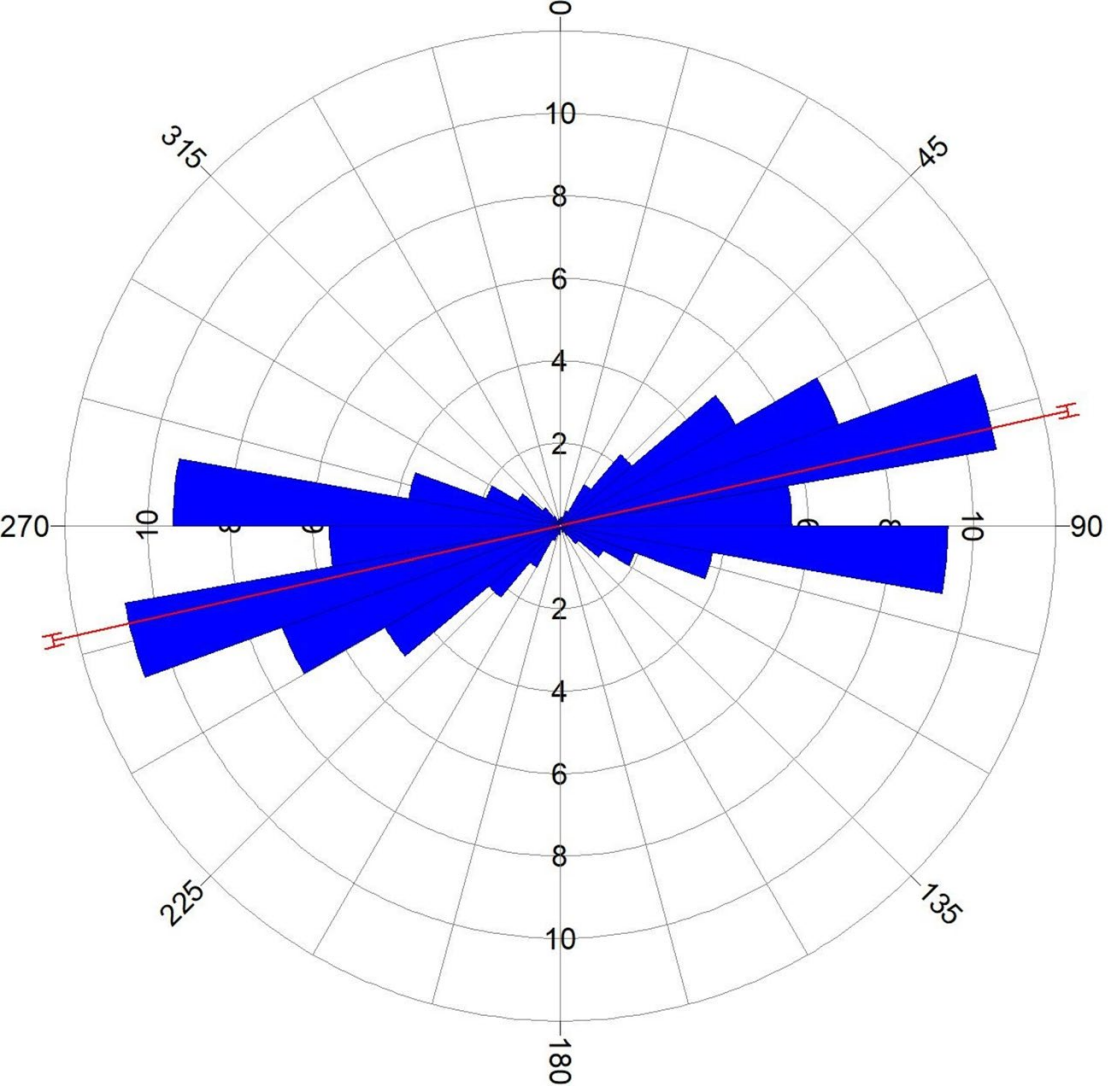
Rose diagram.

To identify areas of structural complexity and deposit occurrence favourability, CETGA was used to assess the texture and locate laterally consistent fissures, such as lineaments along the edges and ridges of magnetic bodies. The CETGA was employed on the RTE grid. A step-by-step process was used to estimate magnetic variation using textural analysis; detect any laterally consistent and continuous structures of discontinuity using phase symmetry; amplitude thresholding to suppress background signals and noise to improve lineaments; generate lineaments using skeletonisation (Fig. 9); convert features to smooth the generated lineaments; and, lastly, create a lineament density map that distinguishes regions based on their lineament density (Fig. 10). The lineament density map has been used in several works as a primary indicator for mineralisation^20,27,28,39^. Consequently, a rose diagram was plotted, and it revealed the dominant structural trend to be ENE–WSW followed by ESE–WNW (Fig. 11). The structural signature shows some level of similarity with the analytical signal and the first vertical gradient. Within the study area,^50^ obtained NE–SW and W–E as the dominant trends. The NE–SW fault has been thought to be a result of the Pan-African orogeny^10^. The NE–SW and NNE–SSW trends were reported to be favourable structural controls for gold mineralisation^9^.

### Airborne radiometric data

#### K/Th

**Fig. 12:**
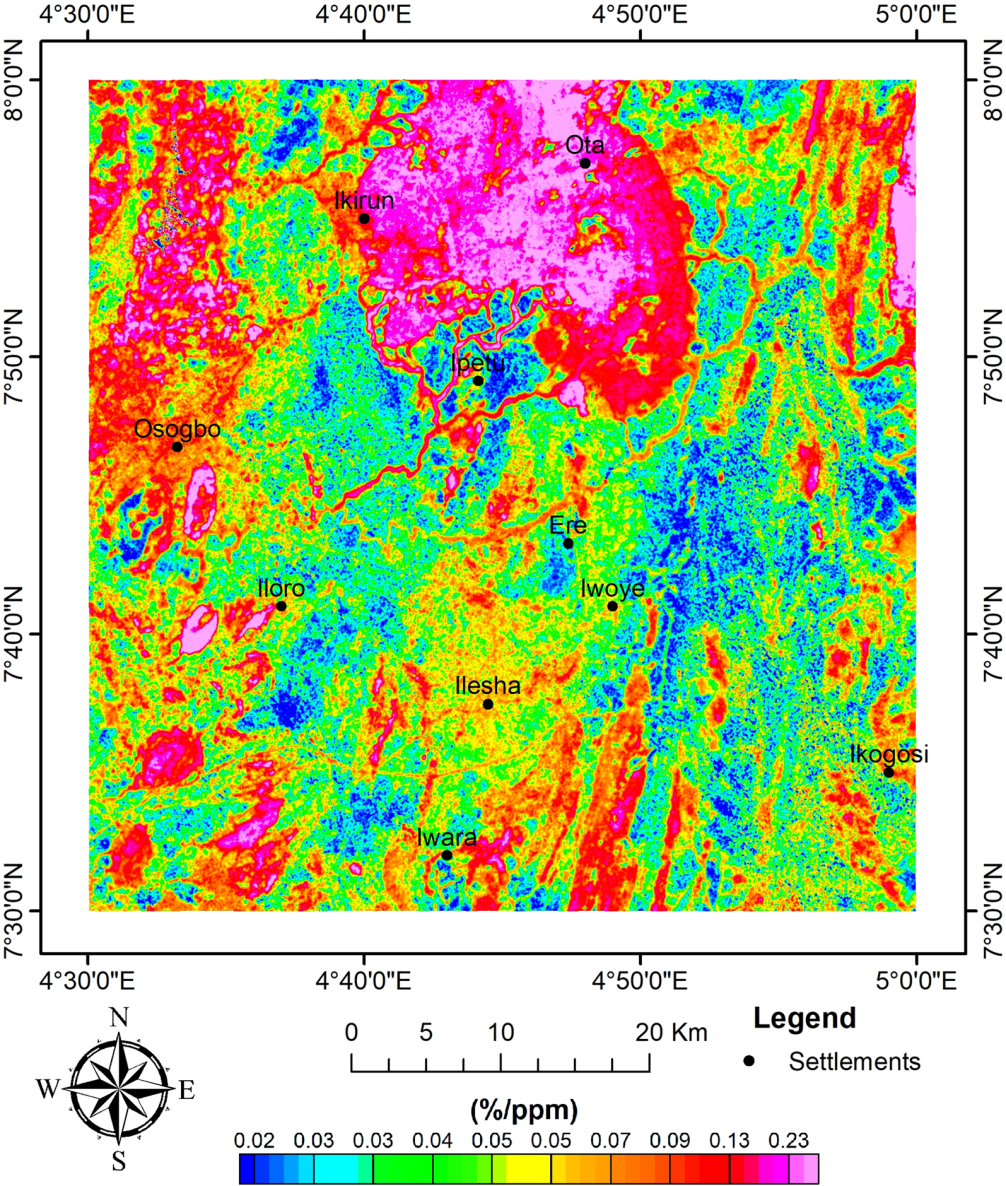
K/Th ratio map of the study site.

The K/Th ratio detected the regions of potassium enrichment, which are unique zones of hydrothermal alterations^14,49^. High K/Th ratios are reliable markers of hydrothermal alteration^48^. The K/Th ratio map (Fig. 12) shows that the alteration zones are characterised by 0.09–0.23%/ppm. However, this shows some level of similarity with the range (0.013–0.362%/ppm) obtained by Ref.^48^. The large K/Th ratio zone at the northern part of the site appears to be a sizeable felsic rock rich in potassium or a metamorphosed felsic rock^16,49^, while the southern part of the study area shows some high K/Th ratio that appears to be structurally controlled.

**Fig. 13:**
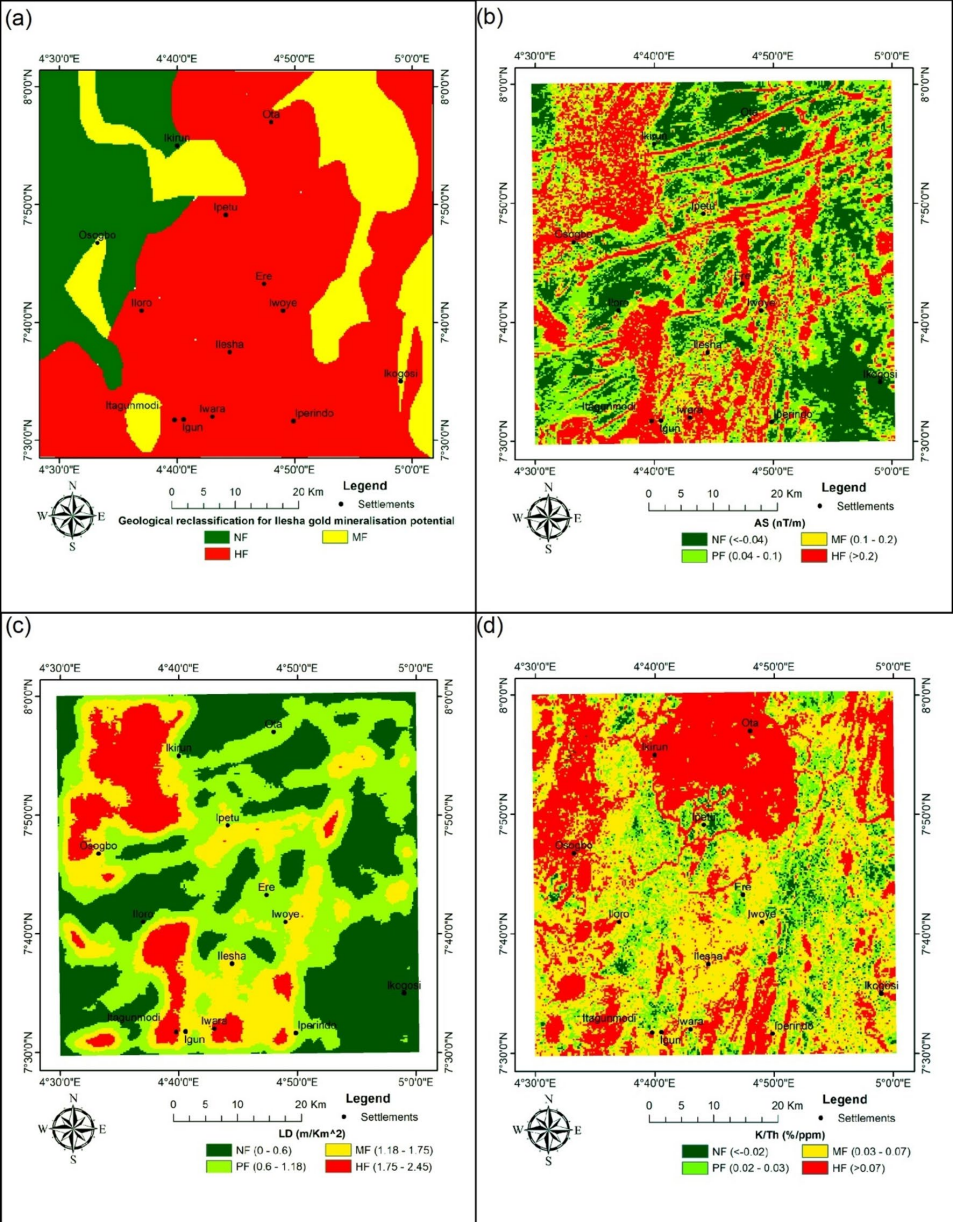
Reclassified maps. (**a**) Geology (**b**) AS (**c**) LD (**d**) K/Th.

The relatively high K/Th ratio can be considered for hydrothermally altered zones. This is because there could be depletion in the radioactive elements due to intense weathering^49^, and this is the case of the known mineralisation host rocks^58^. The results obtained for the K/Th are very similar to those obtained by Ref.^48^. Not much variation was observed using higher-resolution data (as employed in this study) (Fig. 13).

### Weight of AHP

The specialists who created the AHP scale and ranked the criteria according to their professional judgement have a big say in how the gold mineralisation potential is judged in the area. After carrying out a background survey and a review of pertinent literature, five criteria were selected for the study. A pairwise matrix was utilised to acquire the expert's judgement on these criteria, hence providing support for the AHP. The weights obtained from the AHP, as derived from professional judgement, were then used in the weighted overlay algorithm to give each criterion layer a priority.

According to the result of the AHP weight, the parameter "AS, LD, K/Th, and Geology" has an equal cumulative weight index (CWI) value of 0.250 (Table 3). This implies that parameters have equivalent levels of contribution to the consistency outcome. The AHP analysis yielded a cumulative index and random index of 0 and 0.9, respectively. Consequently, a consistency ratio of 0% was obtained, which is most desired^75,76^.

**Table 3:** Results from the AHP for evaluating the favourability of gold mineralisation.

Pairwise Comparison matrix						CWI	Consistency result
	AS	LD	K/Th	F-parameter	1VG		
AS	1	1	1	1	1	0.250	CI = 0 RI = 0.9 CR = 0
LD	1	1	1	1	1	0.250	
K/Th	1	1	1	1	1	0.250	
Geology	1	1	1	1	1	0.250	

### Gold mineralisation potential assessment

**Fig. 14:**
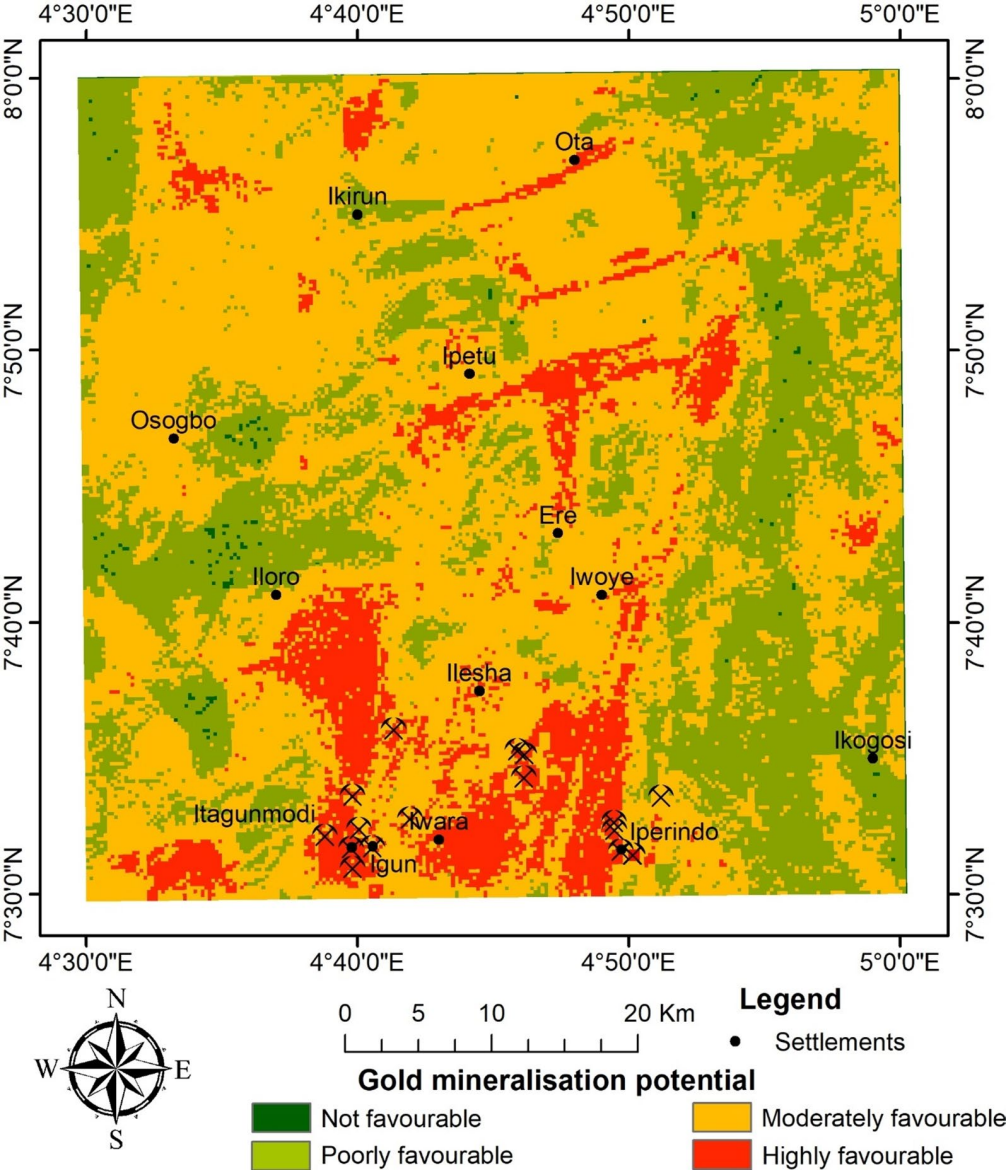
Gold mineralisation potential map in Ilesha schist, southwestern Nigeria.

The AS, LD, K/Th, and lithology were evaluated as rasters. Overlaying four raster layers, each of which corresponded to a criterion mentioned in Table 3, allowed for the construction of a gold mineralisation potential map over the Ilesha schist belt (Fig. 14). Table 4 presents the split of the favourability classes of various areas, expressed in km^2^ and percentages (%). The favourability classes are "Highly favourable," "Moderately favourable," "Poorly favourable," and "Not favourable".

**Table 4:** Distribution of gold mineralisation potential in the Ilesha schist belt, southwestern Nigeria.

Favourability class	Area (km^2^)	Area (%)
Highly favourable	329.11	10.52
Moderately favourable	1889.28	60.39
Poorly favourable	902.87	28.86
Not favourable	7.19	0.23

The highly favourable class covers 329.11 km^2^, which is about 10.52% of the total area of the study site. The moderately favourable class encompasses a much larger area than the highly favourable class, covering a total of 1889.28 km^2^, constituting approximately 60.39% of the entire area. The poorly favourable class appears to cover 28.86% of the area, which is equivalent to 902.87 km^2^. Meanwhile, the "Not favourable" class covers a smaller area of 7.19 km^2^, accounting for 0.23% of the entire area.

The AHP methodologies have been employed in recent studies on appropriateness evaluation to determine the weights of critical criteria^50,80,81^. These investigations have identified important parameters as essential components for modelling. Based on previous research and expert knowledge, the AHP weights assigned a higher priority to each criterion inside the weighted overlay. These weights were from a matrix that had been standardised. The results of the AHP weights showed that the lithology, analytical signal, lineament density, and K/Th ratio all have a significant effect on the gold mineralisation because they are directly linked to the nature of the occurrence of the gold mineralisation under study. The work of Ref.^50^ within the southern portion of the study area utilised lineament density, resistivity data, and geological distribution. However, this study covers a regional part of the Ilesha schist belt, and the focus of this study is tilted toward the style of the gold mineralisation within the study site. Additionally, higher-resolution airborne magnetic and radiometric datasets were employed to unveil the gold mineralisation potential. The metallic association, structural control, and hydrothermal alterations of the gold mineralisation style have shown the relevance of the aforementioned enhancement techniques.

### Optimisation by validation

Validation was made by plotting the coordinates of existing mining sites for correlation purposes to ascertain the effectiveness of the highlighted target zones for gold mineralisation potential. Some mining sites were observed. Additionally, the mining sites observed by Ref.^50^ are remarkable sites within the study area. However, they are all artisanal workings. They are also favoured by the topography of the locations (Fig. 2), where they occur as alluvial deposits. Sixteen of the gold mines were plotted. 94% (i.e., 15 out of 16) of the mines fall within the highly favourable class, while 6% fall within the moderately favourable class. None of the known mine sites fall into the poorly favourable or not favourable class. In summary, all the mine sites fall within the highly and moderately favourable class (i.e., 100% agreement). This shows a high level of accuracy in the prediction. In comparison to the deductions of Ref.^50^, 79% are in agreement, plotting within the high to moderate potential zones. This high percentage of agreement may be a result of contributions from the analytical signal and the radiometric ratios, which have not been integrated in previous works. This indicates that there are potential prospects within the study area that are yet to be unveiled. The northwestern part and other regions delineated to be favourable for gold mineralisation across the study area are relevant targets (Fig. 14).

## Conclusion

The demand for sustainable development goals and the absence of systematic development and organised exploration for gold has prompted this study to integrate magnetic and radiometric datasets with lithology to evaluate the gold mineralisation potential in the Ilesha schist belt. As a result, several signal enhancement techniques were employed to aid data interpretation, including analytic signal, lineament density, and K/Th. With the use of expert opinions, the Analytical Hierarchy Process (AHP) model was used to compare criteria and assign weight values in order to determine the potential of gold mineralisation.

The AS map categorised the area's magnetic potential into low, intermediate, and high. It reveals structures, contacts, and intrusives that could be promising targets for mineralisation. The CETGA delineates the lineament distribution of the area, revealing a dominant trend of ENE–WSW, followed by the ESE–WNW. The K/Th was used to reveal the hydrothermal alteration zones. The analytical signal depth estimate of magnetic sources reveals 63.17–629.47 m of shallow to deeper sources, while the Euler deconvolution delineated a depth range of 47.32–457.22 m. The delineated hydrothermal alteration zones, high lineament density, and highly magnetic zones are significantly associated with active gold mines. The gold mineralisation potential map reveals 10.52% of the area to be highly favourable, 60.39% of the area to be moderately favourable, while the poorly favourable class covers the larger part of the study site with an aerial coverage of 28.86%. The unfavourable class covers the least portion (0.23%). The result was optimised by using known mines as a validation tool. 94% (i.e., 15 out of 16 mining sites) plotted within the high mineralisation potential class. The higher level of accuracy may be attributed to the higher resolution of datasets used and/or contributions from the analytical signal and the K/Th ratio.

Policymakers and stakeholders should prioritise the highly favourable class for further exploration and development. Putting these strategies into effect will aid in the optimisation of gold production in the study area and, of course, promote sustainable mineral development in Nigeria.

## Data Availability

The high-resolution airborne magnetic and radiometric data can be obtained from the Nigerian Geological Survey Agency. However, the corresponding author can make provision of the data based on a reasonable request.
